# An open-label trial to compare exposures of subcutaneous long-acting injectable use olanzapine (TV-44749) versus oral olanzapine in adults with schizophrenia (ORBIT)

**DOI:** 10.1192/j.eurpsy.2026.10534

**Published:** 2026-07-31

**Authors:** A. Merenlender Wagner, K. Shulman, R. Gidron-Budovsky, A. Elgart, A. Jehassi, N. Sharon, R. Apolinario, G. Davis, K. Duma, M. Suett, D. Gutman, L. Rabinovich-Guilatt

**Affiliations:** 1 Clinical Pharmacology, Innovative Medicines & Global Clinical Development, Teva Pharmaceutical Industries Ltd., Netanya, Israel; 2 Innovative Medicines & Global Clinical Development, Teva Pharmaceutical Industries Ltd., West Chester, PA, United States; 3 Innovative Medicines & Global Clinical Development; 4 Global Statistics, Global Specialty Research & Development, Teva Pharmaceutical Industries Ltd., Netanya, Israel; 5 Produtos Farmacêuticos, Lda, Global Regulatory Affairs – Innovative Medicines, Teva Pharmaceutical Industries Ltd., Porto-Salvo, Portugal; 6 Global Innovative Medicines & Biosimilars Clinical Operations, Teva Pharmaceutical Industries Ltd., West Chester, PA, United States; 7 Global Medical Affairs, Teva UK Limited, Harlow, United Kingdom; 8 Innovative Medicines Research and Development, Teva Pharmaceutical Industries Ltd., Netanya, Israel

## Abstract

**Introduction:**

TV-44749 is an investigational, long-acting, subcutaneous (SC) formulation combining olanzapine (OLZ) and an innovative copolymer-based drug delivery technology in a long-acting injectable (LAI) suspension intended to address the limitations of oral and intramuscular OLZ. Phase 1 trial results showed that TV-44749 exhibited a favorable extended-release pharmacokinetic (PK) profile and that once-monthly doses of 318, 425, and 531 mg yield comparable OLZ exposures corresponding to oral daily doses of 10, 15, and 20 mg, respectively. These results guided dose selection for the phase 3 SOLARIS trial (NCT05693935) in adults with schizophrenia (SZ). Through the double-blind, placebo-controlled, acute treatment phase (Period 1) of SOLARIS, all three TV-44749 doses resulted in significantly greater efficacy. The long-term safety data from SOLARIS (Period 2) demonstrated that TV-44749 was generally well tolerated. Across the clinical development of TV-44749, no suspected or confirmed post-injection delirium/sedation syndrome (PDSS) events were reported (3990 TV-44749 injections).

**Objectives:**

The primary objective of the ORBIT trial is to assess the relative bioavailability of TV-44749 compared with EU-registered oral OLZ in adults with SZ, and support PK bridging between the two formulations. Secondary objectives include additional PK, safety, and tolerability endpoints. Trial design was based on the European Medicines Agency guidelines (Document: EMA/CHMP/EWP/280/96 Rev1) and TV-44749 phase 1 PK trial results.

**Methods:**

ORBIT is an ongoing phase 1, 21-week, multicenter, open-label, multiple-dose, single-sequence trial in people living with SZ (NCT06315283). Adults (aged 18–64 years) clinically stable on oral OLZ 20 mg/day over ≥4 weeks before screening and not currently on other antipsychotic treatments were recruited in the US and EU. In treatment Period 1, participants receive oral OLZ 20 mg daily for 7 days. Treatment Period 2 comprises three TV-44749 531 mg once-monthly administrations. The primary endpoint is the OLZ exposure ratio between TV-44749 and oral OLZ administration over a 28-day dosing interval. Secondary endpoints include minimum plasma concentration at end of dosing interval and maximum observed plasma drug concentrations for both treatments. Safety and tolerability are also being assessed.

**Results:**

Recruitment is complete, with anticipated trial completion by September 2025.

**Conclusions:**

When completed, ORBIT trial results will add to the body of evidence, including modeling and simulation, to support establishing a PK bridge between TV-44749 and oral OLZ, and provide additional PK, safety, and tolerability data. TV-44749 may become the first LAI OLZ formulation that avoids the Risk Evaluation and Mitigation Strategy (REMS) associated with PDSS in adults with SZ.

**Disclosure of Interest:**

A. Merenlender Wagner Employee of: Teva, K. Shulman Shareholder of: Teva, Employee of: Teva, R. Gidron-Budovsky Employee of: Teva, A. Elgart Shareholder of: Teva, Employee of: Teva, A. Jehassi Employee of: Teva, N. Sharon Employee of: Teva, R. Apolinario Shareholder of: Teva, Employee of: Teva, G. Davis Shareholder of: Teva, Employee of: Teva, K. Duma Employee of: Teva, M. Suett Shareholder of: Teva, Employee of: Teva, D. Gutman Shareholder of: Teva, Employee of: Teva, L. Rabinovich-Guilatt Employee of: Teva

